# Idiopathic cholesterol pericarditis in a dog presenting with cardiac tamponade

**DOI:** 10.1093/jvimsj/aalag219

**Published:** 2026-09-27

**Authors:** Berry Wong, Shamanthi Shankar, Elpida Sarvani, Alexander Civello, Jessie R Payne

**Affiliations:** Small Animal Referral Hospital, Langford Vets, University of Bristol, Stock Lane, Langford BS40 5DU, United Kingdom; AURA Veterinary, 70 Priestley Road, Guildford GU2 7AJ, United Kingdom; Small Animal Referral Hospital, Langford Vets, University of Bristol, Stock Lane, Langford BS40 5DU, United Kingdom; Willows Veterinary Centre and Referral Service, Highlands Road, Shirley, Solihull B90 4NH, United Kingdom; Diagnostic Laboratories, Langford Vets, University of Bristol, Stock Lane, Langford BS40 5DU, United Kingdom; Veterinary Pathology Group Histology, 540 The Crescent, Bristol Business Park, Filton, Bristol BS16 1EJ, United Kingdom; Small Animal Referral Hospital, Langford Vets, University of Bristol, Stock Lane, Langford BS40 5DU, United Kingdom

**Keywords:** cholesterol crystals, chronic pericardial effusion, hypothyroidism, rheumatoid arthritis, tuberculosis

## Abstract

A 7-year, 6-month-old, female neutered, Pug dog presented with lethargy, hyporexia, polydipsia, abdominal distension, and dyspnea. Point-of-care ultrasonography identified tricavitary effusions and cardiac tamponade. Pericardiocentesis yielded 83 mL of sanguineous scintillating fluid. Clinical examination, bloodwork, echocardiography, electrocardiography, thoracic and abdominal computed tomography scans, and pericardial fluid analysis including culture and sensitivity did not identify any cardiovascular, endocrine, neoplastic, or infectious causes of the pericardial effusion. Pericardial fluid analysis identified high cholesterol concentrations and numerous cholesterol crystals on cytology, supporting a diagnosis of cholesterol pericarditis. Subtotal pericardiectomy was performed 7 weeks later to address pericardial and pleural effusions. Histopathological pericardial biopsies identified pericarditis with no neoplastic tissue detected. A pleural port was placed 4 months later to manage recurrent pleural effusion. Elective euthanasia was performed 10 weeks later citing a compromised quality of life from increasingly frequent bouts of dyspnea. This is a case report of a dog with idiopathic cholesterol pericarditis presenting with cardiac tamponade.

A 7-year, 6-month-old, female neutered, Pug dog presented with a 2-day history of lethargy, hyporexia, and polydipsia, and a 1-day history of progressive abdominal distension and dyspnea. Before this examination, the owner had no clinical concerns; the dog had no known exposure to toxic substances, nor was it receiving any routine medications. Apart from a single self-resolving episode of lameness 3.5 years before referral, there was no relevant orthopedic history.

The dog was overweight at 12 kg, with a body condition score of 7/9. Thoracic auscultation revealed tachycardia at 160 beats per minute with a regular rhythm, quiet heart sounds, and no audible heart murmur. Respiratory rate was 36 breaths per minute, with moderately increased respiratory effort. Otherwise, no abnormalities were detected on clinical examination and there was no evidence of lameness.

Thoracic and abdominal point-of-care ultrasonography identified moderate volumes of pericardial effusion (PE) and pleural effusion, and a small amount of peritoneal effusion. There was collapse of the right atrial wall consistent with cardiac tamponade, and the heart was subjectively underloaded (Figure 1). Pericardiocentesis and thoracocentesis yielded 83 mL of sanguineous scintillating fluid and 155 mL of serosanguinous fluid, respectively. The peritoneal effusion was not sampled because of the scant volume present.

**Figure 1:**
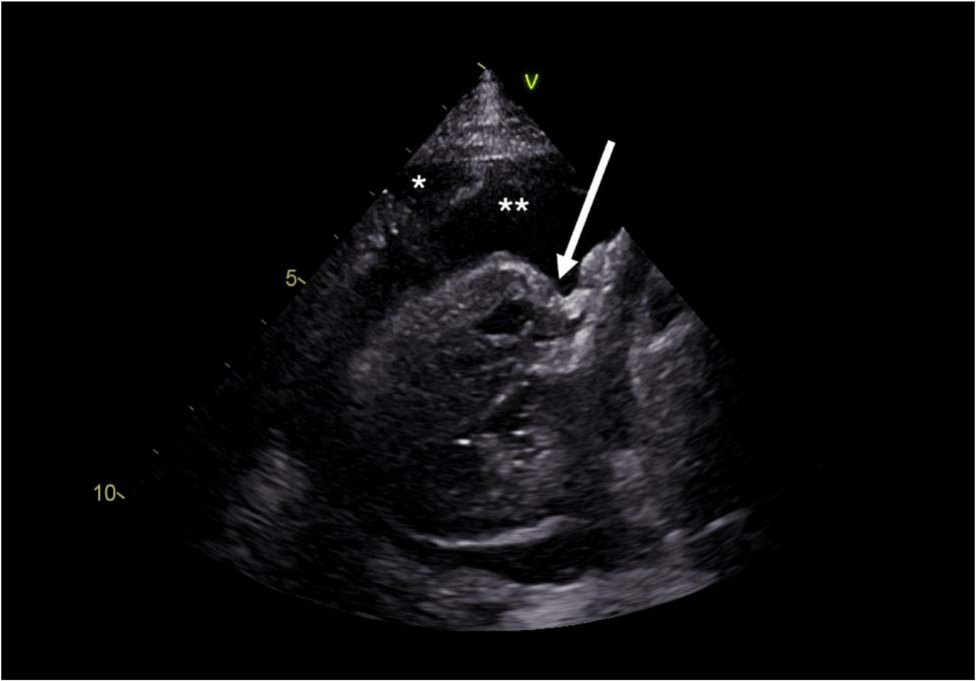
Echocardiography demonstrated pleural effusion (^*^) and pericardial effusion (^**^). The arrow is pointing at the collapse of the right atrial wall.

Echocardiography was performed after centesis. No overt evidence of cardiac neoplasia was detected. The tip of the right auricle was considered normal. There was evidence of myxomatous mitral valve disease with borderline left atrial enlargement but without left ventricular enlargement (left atrial to aortic root ratio 1.55, ref < 1.60; end-diastolic left ventricular internal diameter normalized to body weight 1.16, ref < 1.70), American College of Veterinary Internal Medicine (ACVIM) stage B1.^1^ There was a low probability of pulmonary hypertension,^2^ based on a tricuspid regurgitation velocity of 2.5 m/s (ref < 3.0 m/s), but subjective mild dilation of the pulmonary artery. The right atrium and right ventricle were normal in size. There was only subtle variation in mitral inflows associated with respiration, making the presence of effusive constrictive physiology much less likely. There was no evidence of any congenital abnormalities. Simultaneous electrocardiography identified consistent sinus rhythm.

Hematology and biochemistry, including total thyroxine, canine thyroid-stimulating hormone, free thyroxine, troponin I, and basal cortisol, did not reveal a cause for the PE and results were not consistent with hypothyroidism, hypoadrenocorticism, or hyperadrenocorticism. Viscoelastic testing revealed no evidence of hypercoagulability or hypocoagulability. Urinalysis was not performed.

No relevant abnormalities were detected on thoracic and abdominal computed tomography scans. There was a focal, ill-defined region of heterogenous contrast enhancement at the tip of the right auricle, which did not appear as a distinct mass lesion. The cranial mediastinal lymph nodes were prominent and the splenic lymph node was enlarged. These enlargements were considered to be most consistent with reactivity in the lymph nodes, although metastatic disease could not be excluded.

Pericardial fluid analysis was not evidently lipid-rich macroscopically. An accurate automated total nucleated cell count was not possible because of interference in both channels on the analyzer (Siemens, Advia 2120). On cytological examination, high numbers of cells were present, chiefly comprising of atypical mononuclear cells, often in epithelioid arrangements, and suspected reactive mesothelial cells. Mesothelial hyperplasia was suspected, although malignant neoplasia (mesothelial or epithelial) could not be completely excluded. Frequent macrophages and neutrophils, and fewer lymphocytes were also seen, suggestive of inflammation. The fluid hematocrit, hematoidin crystals, and hemosiderophages indicated a hemorrhagic component to the effusion. There were numerous, clear, rectangular, flat crystals, that sometimes had a notched appearance, compatible with cholesterol crystals (Figure 2). Based on this finding, fluid cholesterol and triglyceride levels were measured, with both greater than their respective serum concentrations (fluid cholesterol 5.6 mmol/L, serum cholesterol 4.5 mmol/L; fluid triglycerides 1.62 mmol/L, serum triglycerides 1.43 mmol/L), compatible with cholesterol pericarditis (CP).^3^

**Figure 2:**
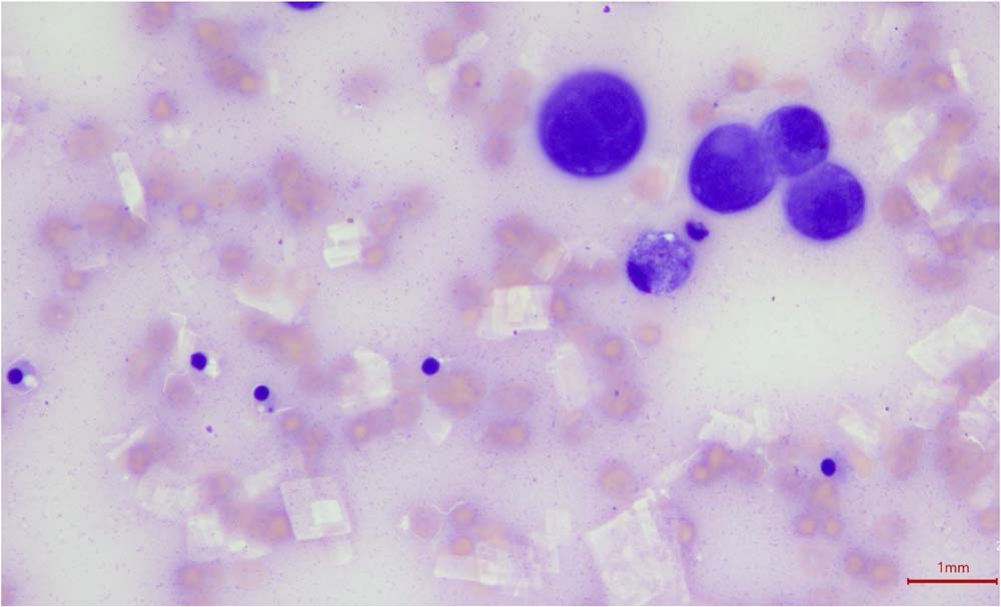
Photomicrograph of the cytologic preparation of pericardial fluid from the dog showing numerous clear, rectangular, flat crystals. Modified Wright stain, 50× (oil) objective.

Pleural fluid analysis revealed a protein-rich transudate with atypical mesothelial cells that were suspected to be reactive. Both pericardial and pleural fluids did not yield any growth upon aerobic and anaerobic bacterial culture.

The dog was discharged after resolution of clinical signs but represented 7 weeks later with similar signs and again had pericardial and pleural effusions. Subtotal pericardiectomy was performed and histopathological evaluation (Figure 3) was subsequently carried out. Histopathology of the pericardium demonstrated fibrosis and loss of mesothelium diffusely affecting the inner lining, mild neutrophilic and lymphocytic inflammation, and evidence of chronic hemorrhage in the form of hemosiderophages. Occasional aggregates of macrophages laden with cytoplasmic lipid vacuoles were present in the pericardial fat. There was no evidence of neoplasia.

**Figure 3:**
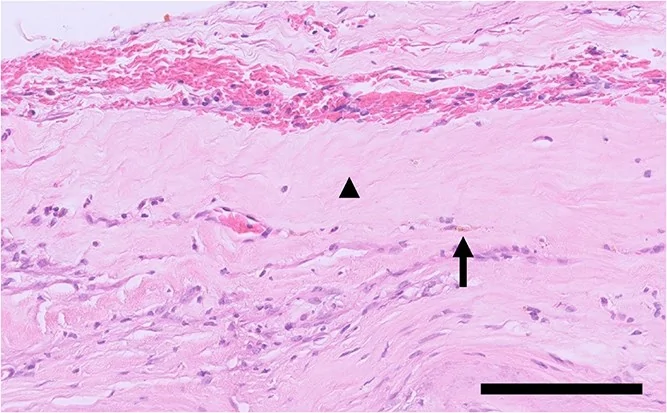
Histopathology of pericardial biopsy. Fibrosis affecting the internal surface of the pericardium (arrow head). Rare deposits of hemosiderin are present (arrow). Hematoxylin and eosin 200× magnification. Scale bar = 200 microns.

A pleural port was placed 4 months later to address recurring dyspneic episodes secondary to pleural effusion, previously managed with thoracocentesis. Repeat echocardiography showed no evidence of neoplasia. Histopathology of the pleura demonstrated chronic, neutrophilic, lymphocytic, and plasmacytic pleuritis, with mesothelial hypertrophy and hyperplasia, and lymphatic colonization.

The dog was euthanized 10 weeks later, 8.5 months after the original diagnosis, due to a compromised quality of life from increasingly frequent bouts of dyspnea.

## Discussion

Cholesterol pericarditis remains rare ever since the first report describing a human patient with chronic PE of “scintillating gold paint appearance” was published over 100 years ago.^4^ Hypothyroidism, tuberculosis, rheumatoid arthritis, and hyperlipidemia are associated with CP in humans,^3^^,^^5–10^ although most cases remain idiopathic.^3^^,^^11^ There is only one case report in the veterinary literature describing a dog that presented with cholesterol-based PE and aortic thromboembolism associated with hypothyroidism.^12^ This is a case report of a dog with idiopathic CP.

In human literature, CP is a rare pericardial disease characterized by chronic PE with high cholesterol concentrations, with or without the formation of cholesterol crystals.^3^^,^^5^ While the clinical history and presentation of this dog were suggestive of acute cardiac tamponade, previous episodes of acute PE without clinical signs were possible and therefore an acute-on-chronic etiology of the fluid characteristics could not be ruled out. The pathophysiology of CP has yet to be elucidated. It is hypothesized that repeated episodes of acute pericarditis cause PE, thickening of the pericardium, and impaired absorptive capacity. As the PE becomes chronic, cholesterol in the pericardial fluid becomes more concentrated, less soluble, and precipitates, thereby further exacerbating PE.^3^^,^^6^ The color of PE in humans with CP has been variable, although a scintillating appearance similar to this dog’s pericardial fluid is more often seen, attributed to its cholesterol crystals content.^3^ This dog’s histopathology results are also similar to common histopathological findings of pericardial biopsies in humans with CP: chronic pericarditis, thickening, fibrosis, and cellular infiltration.^3^ It is worth noting that the absence of cholesterol deposits or clefts on histopathology does not exclude a diagnosis of CP.^3^^,^^12^ There are currently no veterinary guidelines specifying a cholesterol cut-off value in pericardial fluids to classify PE as CP. Pericardial fluid cholesterol levels greater than 1.8 mmol/L with associated cholesterol crystals visualized on microscopy are reported as a diagnostic aid in humans.^5^ Based on this, a diagnosis of CP was made in this dog.

This dog was investigated for diseases that have been associated with CP in people, with no evidence of these diseases identified. Hyperlipidemia was considered as a cause of the CP. Hyperlipidemia itself does not directly lead to the development of clinical signs, but clinical signs can develop due to diseases associated with hyperlipidemia, which might include hepatobiliary disease, pancreatitis, and insulin resistance.^13^ Cholesterol pericarditis is linked to hyperlipidemia in humans^10^ but whether this is the case in dogs remains to be proven. This dog had a normal serum cholesterol and serum triglycerides was only mildly above the reference interval, therefore hyperlipidemia was not considered to be the primary cause of her PE. The dog’s hypertriglyceridemia could be attributed to its body condition. Overweight hypertriglyceridemic dogs exhibit greater oxidative stress^14^ and inflammation, which could have had a contributory role towards the PE.

The presence of hyponatremia and hyperkalemia, coupled with a calculated Na:K ratio of < 27:1, initially raised suspicion of underlying mineralocorticoid deficiency that might have decreased circulatory volume and contributed to cardiac compromise. However, a basal cortisol result of > 55 nmol/L after stabilization of the dog made a diagnosis of hypoadrenocorticism less likely in this case.^15^ Pericardial effusion, in general, can be caused by a wide spectrum of disorders, with neoplastic disease reported as the most common cause in dogs.

Optimal treatment of CP should not be different from that of chronic PE without increased cholesterol, and pericardiectomy is often necessary for most dogs if it is recurrent.^3^ Subtotal pericardiectomy remains the treatment of choice for recurrent PE without an identifiable mass, especially should subsequent constrictive pericarditis be limited to the parietal pericardium and therefore confer a better prognosis than the visceral pericardium being affected.^16^ However, as demonstrated by the recurrent episodes of pleural effusion and subsequent dyspnea in this case, subtotal pericardiectomy is not the definitive treatment for CP, particularly if constrictive pericarditis develops. Dogs like this might continue to produce effusion since the visceral layer of the pericardium and the cardiac epicardium are synonymous and is therefore not removed during surgery. Despite the use of nonsteroidal anti-inflammatory drugs, colchicine, and immunosuppressive drugs in humans with CP,^7^^,^^11^ the therapeutic benefit has not been proven. Therefore, these drugs were not utilized in this dog. Treatment of hyperlipidemia in dogs involves dietary management, with subsequent drug therapy considered in cases of hypertriglyceridemia above 5.5 mmol/L and hypercholesterolemia above 13 mmol/L,^13^ neither of which was true for this dog.

Information on CP in human literature is limited to case reports^4–9^^,^^11^ and a small case series,^3^ with outcomes varying from death due to cardiac tamponade to survival documented 6 years postoperatively,^3^ highlighting the need for extended follow-up of more dogs to better quantify long-term outcomes. Comparison of this dog with the other reported dog with CP and aortic thromboembolism is challenging.^12^ This is because the previously reported case identified a presumptive underlying cause for CP. Therefore, treatment of such primary cause would have likely led to a more favorable outcome compared to the idiopathic nature of this dog’s CP. The dog described here had a demonstrably poorer outcome. Our case had recurrent episodes of pleural effusion and clinical deterioration prompting euthanasia 8.5 months after the original diagnosis whereas the previously reported case was documented to be clinically well and alive 18 months after discharge.

This report describes a case of idiopathic CP, a rare form of PE, and highlights the possible risk factors associated with CP. In dogs with recurrent PE and chronic inflammatory conditions, CP should be considered as a differential and risk factors should be investigated to treat any underlying disease. Subtotal pericardiectomy can be considered to alleviate clinical signs of cardiac tamponade and can prevent recurrent episodes of PE and constrictive pericarditis. While the outcome of this case was poor, the prognosis for idiopathic CP is yet to be determined.

## Off-label antimicrobial use declaration

The authors declare no off-label use of antimicrobials.

## Institutional animal care and use committee or other approval declaration

The authors declare no IACUC or other approval was needed.

## Abbreviations

CP: cholesterol pericarditis
PE: pericardial effusion
